# Comprehensive meta-analysis of QTL and gene expression studies identify candidate genes associated with *Aspergillus flavus* resistance in maize

**DOI:** 10.3389/fpls.2023.1214907

**Published:** 2023-07-18

**Authors:** Niranjan Baisakh, Eduardo A. Da Silva, Anjan K. Pradhan, Kanniah Rajasekaran

**Affiliations:** ^1^ School of Plant, Environmental and Soil Sciences, Louisiana State University Agricultural Center, Baton Rouge, LA, United States; ^2^ Department of Agriculture, Federal University of Lavras, Lavras, Brazil; ^3^ Food and Feed Safety Research Unit, Southern Regional Research Center, United States Department of Agriculture - Agricultural Research Service (USDA-ARS), New Orleans, LA, United States

**Keywords:** aflatoxin, co-expression network, genomic region, maize, marker, Meta-QTL, microarray, RNA-seq

## Abstract

Aflatoxin (AF) contamination, caused by *Aspergillus flavus*, compromises the food safety and marketability of commodities, such as maize, cotton, peanuts, and tree nuts. Multigenic inheritance of AF resistance impedes conventional introgression of resistance traits into high-yielding commercial maize varieties. Several AF resistance-associated quantitative trait loci (QTLs) and markers have been reported from multiple biparental mapping and genome-wide association studies (GWAS) in maize. However, QTLs with large confidence intervals (CI) explaining inconsistent phenotypic variance limit their use in marker-assisted selection. Meta-analysis of published QTLs can identify significant meta-QTLs (MQTLs) with a narrower CI for reliable identification of genes and linked markers for AF resistance. Using 276 out of 356 reported QTLs controlling resistance to *A. flavus* infection and AF contamination in maize, we identified 58 MQTLs on all 10 chromosomes with a 66.5% reduction in the average CI. Similarly, a meta-analysis of maize genes differentially expressed in response to (a)biotic stresses from the to-date published literature identified 591 genes putatively responding to only *A. flavus* infection, of which 14 were significantly differentially expressed (−1.0 ≤ Log2Fc ≥ 1.0; *p* ≤ 0.05). Eight MQTLs were validated by their colocalization with 14 *A. flavus* resistance-associated SNPs identified from GWAS in maize. A total of 15 genes were physically close between the MQTL intervals and SNPs. Assessment of 12 MQTL-linked SSR markers identified three markers that could discriminate 14 and eight cultivars with resistance and susceptible responses, respectively. A comprehensive meta-analysis of QTLs and differentially expressed genes led to the identification of genes and makers for their potential application in marker-assisted breeding of *A. flavus*-resistant maize varieties.

## Introduction

Aflatoxin (AF) contamination in food and feed crops such as maize, peanuts, cottonseed, and tree nuts, caused by *Aspergillus flavus*, continues to be a persistent problem that compromises food safety and marketability worldwide, especially in developing countries. AF are potent carcinogens, and their contamination in food is one of the major causes of liver cancer. AF levels are strictly monitored in food and feed by the Food and Drug Administration (FDA) and European agencies where American food products are imported. Strict enforcement of action levels for AF in over 120 countries places an adverse burden on growers and food processors because of significant economic losses resulting from the decreased value of contaminated commodities (Bedre et al., 2015). Estimates reveal direct annual crop revenue losses in the US in the tens of millions of dollars, and the losses are exacerbated to hundreds of millions of dollars during severe drought years.

Maize is a major food and feed crop grown worldwide and is highly susceptible to *A. flavus* infection. Abiotic stressors such as drought significantly increase AF contamination in maize (Kebede et al., 2012; Fountain et al., 2014). Computer model-based prediction projects that alterations in environmental conditions due to climate change could lead to a significant increase in AF contamination in maize resulting in an estimated annual loss of $50 million to $1.7 billion to the US maize industry (Mitchell et al., 2016). In 2013, economic losses in the US resulting from *A. flavus* alone in maize were estimated to be $686.6 million (Mitchell et al., 2016). Total costs attributable to AF contamination are much higher when factors such as sampling and testing, destruction and disposal, and human and animal health effects are accounted for.

The most efficient and practical approach to reducing pre-harvest AF contamination in maize is the development of resistant lines. There is a dire need for AF-resistant maize germplasms that will also possess resistance to other mycotoxins such as fumonisin and tolerance to abiotic stresses such as drought. Three types of *A. flavus* resistance mechanisms, such as *in vitro* seed colonization, preharvest aflatoxin contamination, and aflatoxin production/accumulation, have been reported in different genetic backgrounds of crops, yet there is no report of a single genotype possessing all three resistance mechanisms (Pandey et al., 2019). Natural resistance to AF contamination in maize is a complex multigenic trait, and therefore, it is difficult and slow to introgress AF resistance traits into agronomically superior commercial varieties. Genomics-enabled marker-assisted breeding (MAB) can facilitate the development of AF-resistant maize germplasm suitable for a specific ecogeographic system. A primary requisite for MAB is the identification and validation of genes and linked diagnostic molecular markers associated with AF resistance. A large number of QTLs and markers associated with AF resistance in maize have been reported from multiple biparental mapping and genome-wide association studies (GWAS) (Soni et al., 2020). Resistance to aflatoxin accumulation explained by an individual QTL has not been more than 20% (Warburton and Williams, 2014), although a QTL explaining up to 41% variance was reported by Mideros et al. (2014). Yet, molecular breeding for the development of AF resistance in maize remains to be realized. This is because most of the markers identified for AF resistance in maize emanate from biparental QTL mapping, which is strongly influenced by experimental design, the genetic background of parents, the type and size of mapping population, the growing environment, the choice and density of markers, and the statistical methods used for analysis (Zhao et al., 2018). Moreover, QTLs detected using different mapping populations with diverse genetic backgrounds under different environmental conditions with often low-resolution genotyping and inconsistent phenotyping explain low and varying phenotypic variance with low logarithm of odds (LOD) scores, which create uncertainty for their introgression by MAB (Price, 2006). The efficacy of the QTLs in MAB is further negated by undesirable epistatic and modifier effects of different genetic backgrounds, which are important for QTL stability (Swamy et al., 2011). Also, the lack of fine mapping of these QTLs with large confidence intervals (CI) hinders the application of these overlapping genomic regions in breeding programs (Zhao et al., 2018).

An alternative approach to fine mapping is to leverage previously identified QTLs from multiple studies through meta-QTL (MQTL) analysis. MQTL analysis is a fast-emerging, effective computational technique that precisely combines previously reported QTLs to identify true QTLs with refined positions by reducing the CI of overlapping QTLs on a reconstructed consensus map (Goffinet and Gerber, 2000; Veyrieras et al., 2007; Sosnowski et al., 2012). Meta-analysis sequentially combines QTLs at a 95% CI reported from different studies where the Akaike Information Criterion (AIC)-based model simulation is performed to determine the number of actual QTLs for the trait of interest (Veyrieras et al., 2007). The MQTLs with clustering of a high number of initial QTLs with the smallest CI and a consistent, major effect on target trait(s) allow the identification of promising trait-linked markers that can be effectively utilized in MAB programs upon their validation in a set of germplasm accessions (Soriano and Alvaro, 2019). Moreover, the identification of accurate locations of the MQTLs facilitates mining the of candidate genes from the available physical map. Functional analysis of these candidate genes can narrow down to the actual gene(s) that will have a direct or indirect effect on the desired traits.

Several studies have identified QTLs for various traits for mining the candidate genes in different crop plants, including maize for traits such as flowering time (Chardon et al., 2004; Wang et al., 2016), popping traits (Kaur et al., 2021), grain moisture content and dehydration (Xiang et al., 2012; Wang et al., 2022), ear rot resistance (Xiang et al., 2012), and abiotic stress tolerance (Zhao et al., 2018; Liu et al., 2019; Sheoran et al., 2022). These studies focused on meta-trait (more than one trait), which are functions of several component traits, and therefore such analysis is not able to conclusively describe the genetic and molecular mechanisms underlying the component traits (Liu et al., 2020). Until now, there have been only two MQTL studies reported in maize for AF and *Aspergillus* ear rot resistance (Xiang et al., 2010; Mideros et al., 2014).

Similarly, several transcriptome studies have been conducted in maize to identify differentially expressed genes with potential roles in the resistance response in maize-*A. flavus* interaction (Luo et al., 2011; Kelley et al., 2012; Dolezal et al., 2014; Shu et al., 2017; Han et al., 2020; Musungu et al., 2020; Liu et al., 2021). However, a meta-analysis of the transcriptome data is lacking in maize. In the present study, a comprehensive meta-analysis of previously published QTLs and genes with putative roles in AF resistance response in maize was conducted to identify candidate causal QTLs/genes and linked markers.

## Materials and methods

### Curation of published literature on QTLs and gene expression and creation of a database

Published literature on QTL mapping for aflatoxin accumulation in maize was curated through database searches using the keywords, viz., QTL, metaQTL, maize, *Aspergillus flavus*, and aflatoxin, using Web of Science (http://apps.webofknowledge.com) and Google search engines. Altogether, 16 reports on separate experiments based on different mapping populations were available on QTL analysis of the AF resistance traits in maize. These QTL studies were scanned for methods of QTL mapping, parents, type and size of mapping population and molecular markers, genetic map, QTL position, peak marker of QTL, marker/confidence interval (CI), LOD score of QTLs, and phenotypic variance explained (PVE or *R* ^2^) by the QTLs. A LOD score of 3.0 was uniformly assumed for experiments that did not provide the LOD value for the QTLs. For studies that did not provide CI for QTLs, the 95% CI was calculated as described by Darvasi and Soller (1997) and Guo et al. (2006). Most of the studies reported QTLs from experiments involving individual environments (locations), years, and overall based on data from multiple environments/years. Therefore, each QTL reported for each year and location in the studies was treated as an independent QTL. Of these QTL reports, 11 published a genetic map and analyzed their data using composite interval mapping (CIM) and/or multiple interval mapping (MIP) to report QTLs with information on key parameters such as QTL peak and flanking markers, LOD score, and PVE, which were used to perform the meta-analysis (**Supplementary Table S1**).

Similarly, literature available on the gene expression changes in response to several abiotic and biotic stresses in maize was collected following a thorough search for all stress-related studies from both microarray and RNA-seq data available in the NCBI Gene Expression Omnibus (Luo et al., 2008; Luo et al., 2011; Kelley et al., 2012; Shu et al., 2017; Kebede et al., 2018; Han et al., 2020; Musungu et al., 2020; Liu et al., 2021) (**Supplementary Table S2**). All the genes and their relative fold change in expression values were collected and/or deduced using signal intensity, FPKM, or TPM values using methods described earlier (Baisakh et al., 2012; Bedre et al., 2015).

### QTL meta-analysis

#### Construction of consensus map

First, a consensus genetic map was developed using the B73 reference genetic map available in MaizeGDB (https://www.maizegdb.org/data_center/map). The genetic maps and QTL files from published data, as well as the reference map, were uploaded in BioMercator v4.2.3 (De Oliveira et al., 2014), following an InfoMap analysis (Veyrieras et al., 2007) to confirm that all genetic maps shared at least one marker among them and the reference map. The consensus map was created by ConsMap, which calculates the goodness-of-fit value of the maps for each chromosome (Veyrieras et al., 2007; Truntzler et al., 2010). If a common marker was not found between the individual genetic map and the consensus map, a third map was used as a cross-reference based on the marker positions and order.

#### Projection of QTLs

All QTLs with LOD score, *R* ^2^ value, flanking marker positions, and CI from published studies were projected on the consensus genetic map using the QTLProj command, which utilized a simple scaling rule between the original QTL marker interval and the corresponding interval on the consensus map (Veyrieras et al., 2007). The new CI of the projected QTLs was approximated with a Gaussian distribution encompassing the most probable QTL position. Two initial QTLs that did not meet a minimal distance ratio of 0.25 and the *p*-value of homogeneity of flanking markers between the original map and the consensus map at 0.5 were not projected.

#### Meta-analysis of QTLs and genes

Meta-analysis of independent QTLs obtained from different mapping populations, locations/environments, and years was performed using the consensus map with QTL projections using the Meta-analysis command to predict the MQTLs (representative consensus regions). Meta-analysis was performed in two steps. First, QTLs on each chromosome were clustered, assuming their normal distribution around the true location. All possible QTL combinations were tested with maximum clusters (*K*max) set to 15, and the one with the maximum likelihood was selected. The QTL model on each chromosome was selected using the AIC or corrected AIC (AICc, when the QTL sample size was less than six), which returned the number of MQTLs that represent the most significant regions. Next, MQTLs were generated from QTL projection on a consensus map based on the best model with the lowest AIC. Furthermore, the position and 95% CI of the MQTLs were calculated, and the flanking markers for MQTLs were retrieved.

A local database was created with the genes retrieved from the gene expression omnibus that are differentially regulated in maize under various biotic and abiotic stresses (unpublished data). Based on the gene ID comparison, a sub-set of genes with expression changes unique to AF resistance was identified.

### Genes associated with MQTLs

#### Identification of candidate genes in MQTL regions

Genes, along with their available functional annotation, linked to each MQTL within their confidence intervals were retrieved using the genome version in Biomercator 4.2.3, which was built based on the ZmB73_5b genome. Genes common between MQTL intervals and differentially regulated under *A. flavus* (Musungu et al., 2020) were queried against the putative unique genes expressed by *A. flavus* infection to identify candidate genes within MQTLs responsive exclusively to *A. flavus*.

#### Gene ontology and Kyoto Encyclopedia of Genes and Genomes pathway analyses

AgriGO v2.0 (Tian et al., 2017) was used to perform the gene ontology (GO) enrichment analysis for the genes common between MQTLs-linked genes and *A. flavus*-responsive genes to examine the GO terms overrepresented that describe gene products in biological processes, molecular functions, and cellular components. Enrichment for each GO slim term was queried in the gene list to identify related gene entries and GO terms at a hypergeometric significance threshold (FDR < 0.05). Kyoto Encyclopedia of Genes and Genomes KEGG pathway enrichment analysis of the genes was performed using the KEGG mapper (https://www.genome.jp/kegg/mapper/).

#### Expression of candidate *Aspergillus flavus* responsive genes linked to MQTLs

Expression of the unique *A. flavus*-responsive genes in MQTL regions was assessed using the Log_2_-fold change values in inoculated maize tissues relative to uninoculated control reported in the RNA-seq data of Musungu et al. (2020). In addition, the expression of these genes under abiotic stresses such as drought, salt, and the heat was analyzed using qTeller, a comparative RNA-seq expression platform in maizeGDB, which compares expression across multiple data sources in a user-provided gene list. Heat maps with hierarchical clustering of the genes based on their log2 fold-change expression values were built for visualization using the R package pheatmap.

#### Gene co-expression network analysis

The log2 fold-change values of significantly differentially expressed genes (*p* ≤ 0.05) common between MQTLs and Musungu et al. (2020) were used for co-expression network analysis using the R package WGCNA. Clusters (modules) of highly correlated genes (nodes) and intramodular hub genes were identified using the eigen values (Langfelder and Horvath, 2008). Briefly, the soft threshold = 9, according to the criterion of scale-free topology module, was selected to create an adjacency matrix, which was transformed into a topological overlap measure (TOM) matrix to estimate its connectivity of the network, and the weighted adjacency matrix of genes was hierarchically clustered based on dissimilarity among genes. The minimal gene number of 30 and a threshold cut-off of 0.25 were set to identify significant modules. In each intramodular connectivity, the genes with KM value >0.8 were declared as hub genes.

### MQTL validation

A two-way validation was performed for the MQTL identified in the study. The MQTLs and the markers associated with *A. flavus* resistance in maize from five reported GWAS (Farfan et al., 2015; Warburton et al., 2015; Zhang et al., 2016; Han et al., 2020; Bertagna et al., 2021) were compared based on the physical positions to ascertain their proximity. Also, the consensus map of maize was used to locate SSR markers flanking each MQTL and their genomic positions. Primers for 12 SSR markers closest to the MQTLs that colocalized with GWAS SNPs (except MQTL2.4 and MQTL8.2) were custom synthesized at Integrated DNA Technologies Inc. (www.idtdna.com). Based on the reports on the resistance response of maize genotypes to *A. flavus* (Busboom and White, 2004; Brooks et al., 2005; Menkir et al., 2006; Bello, 2007; Alwala et al., 2008; Warburton et al., 2009; Warburton et al., 2011; Willcox et al., 2013; Williams et al., 2015; Brown et al., 2016; Smith et al., 2019; Womack et al., 2020; Castano-Duque et al., 2021; Ogunola et al., 2021), 14 resistant and eight susceptible maize genotypes were selected for MQTL/marker validation.

Maize seeds were germinated in moist filter paper inside an incubator at 28°C. Leaf tissues from 1-week-old seedlings were used for total genomic DNA extraction by a modified CTAB protocol and the DNA quality and quantity were assessed on a 1% agarose gel and nanodrop as described earlier (Khan et al., 2013). Polymerase chain reaction (PCR) was performed using 50 ng genomic DNA following the thermal profile: one cycle of denaturation at 95°C for 5 min, 35 cycles of 95°C for 45 s, 58°C for 45 s, and 72°C for 1 min, followed by a final extension at 72°C for 5 min, and the PCR amplicons were resolved on a 12% polyacrylamide gel using the method described in Khan et al. (2013).

## Results

### QTLs compilation and integrated linkage map construction

A total of 356 QTLs controlling *A. flavus* resistance and/or AF accumulation were reported from 17 studies, with the highest number of QTLs (76) on chromosome 1 and the lowest (13) on chromosome 9 (**Supplementary Table S1**). The average PVE by the QTLs was 7.56%, with a range from 0.004% to 53%. Only 11 studies reporting 278 QTLs that provided adequate relevant information on linkage maps and QTL parameters were used for map projections and subsequent MQTL analysis. The remaining six experiments lacked either genetic maps or molecular markers delimiting the QTLs that were not found in the final integrated linkage map for their use in the downstream investigation. The integrated linkage maps associated with *A. flavus* resistance were assembled from 11 published individual linkage maps and the reference map of maize variety B73 (https://www.maizegdb.org/data_center/map). The integrated linkage map contained 960 markers with a total length of 2,372.9 cM and an average marker density of 2.47 markers/cM (**Supplementary Table S3**).

### Consensus map and QTL projections

The consensus genetic map developed using the integrated linkage map on the B73 reference map with shared markers and the goodness-of-fit value of the genetic maps for each chromosome containing the markers linked to the QTLs from published data consisted of a total of 5,023 loci that included 2,968 SNPs and 2,055 SSR markers (**Supplementary Table S4**; **Supplementary Figure S1**). The consensus map was highly saturated, covering a total length of 2,555.10 cM with an average genetic distance of 0.51 cM between the adjacent markers. Comparison of the consensus map with the physical map obtained from the B73 RefGen_V5b reference genome showed very high collinearity in SSR marker order with 94.8% correlation (**Supplementary Table S4**), which suggested that the consensus map was perfectly ideal for QTL projection. Therefore, 276 out of 278 QTLs, considered out of 356 initial QTLs, were successfully projected on the map.

### MQTL identification and distribution

**Figure 1:**
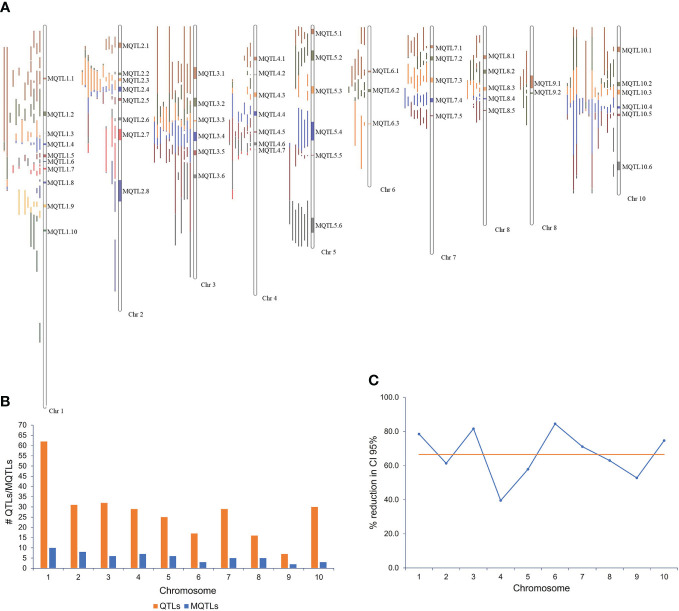
MQTLs associated with resistance to *Aspergillus flavus* and aflatoxin accumulation in maize. **(A)** Fifty-eight MQTLs distributed over 10 maize chromosomes; **(B)** chromosomal distribution of QTLs and MQTLs; **(C)** reduction (%) of confidence interval (CI) of MQTLs relative to the original QTLs.

The meta-analysis of 276 projected QTLs identified a total of 58 MQTLs distributed over all 10 maize chromosomes, with chromosome 1 having the highest number (10), while chromosome 9 registered only two MQTLs (**Table 1**; **Figures 1A, B**). On average, each MQTL accounted for 4.8 original QTLs, with six MQTLs (MQTL2.2, MQTL2.6, MQTL4.3, MQTL5.3, MQTL8.4, and MQTL 10.6) spanning two QTLs, whereas MQTL 1.1 covered 16 original QTLs.

**Table 1:** Detail information on the 58 MQTLs generated with the 276 initial QTLs associated with *Aspergillus flavus* resistance in maize.

Chr.*	Meta-QTL	Phys_start	Phys_end	Position (cM)	CI MQTL	Bin	No. genes	No. QTLs	QTL_id (refer to **Supplementary Table S1**)	Mean *R* ^2^	Avg. CI (QTLS)	% reduction	% reduction by chr.
1	MQTL1.1	40,751,922	42,220,789	56.77	2.01	1.03	33	16	73, 97, 128, 129, 130, 131, 132, 134, 168, 170, 171, 173, 176, 228, 287, 295	6.37	22.53	91.08	
	MQTL1.2	67,582,493	71,265,622	95.00	5.04	1.05	61	8	169, 172, 174, 177, 215, 221, 234, 256	6.68	17.83	71.73	
	MQTL1.3	84,638,887	84,858,121	115.97	0.30	1.05	5	6	85, 229, 253, 254, 343, 345	10.20	10.76	97.21	
	MQTL1.4	92,889,388	94,292,485	128.07	1.92	1.05	9	5	34, 208, 242, 260, 341	10.73	8.83	78.26	
	MQTL1.5	101,527,203	103,938,775	140.58	3.30	1.05	24	3	235, 243, 261	14.72	5.89	43.97	
	MQTL1.6	108,067,680	108,213,835	147.98	0.20	1.05	3	4	33, 216, 245, 257	5.95	6.24	96.79	
	MQTL1.7	112,240,431	113,643,527	154.55	1.92	1.05	15	6	35, 222, 244, 246, 258, 344	9.10	6.26	69.33	
	MQTL1.8	123,176,547	124,733,107	169.62	2.13	1.05	14	3	94, 95, 209	10.07	7.2	70.42	
	MQTL1.9	141,212,186	143,631,066	194.89	3.31	1.06	16	6	98, 99, 133, 270, 271, 346	4.19	13.1	74.73	
	MQTL1.10	161,144,198	162,708,066	221.58	2.14	1.07	27	5	36, 175, 217, 230, 342	3.88	26.1	91.80	78.53
2	MQTL2.1	13,707,326	18,150,443	20.65	5.76	2.02	132	4	4, 135, 178, 183	6.69	11.82	51.27	
	MQTL2.2	38,746,143	40,967,702	51.67	2.88	2.04	42	2	101, 247	6.66	20.35	85.85	
	MQTL2.3	43,297,253	45,811,934	57.76	3.26	2.04	63	10	38, 65, 100, 180, 184, 231, 255, 262, 263, 348	7.90	17.17	81.01	
	MQTL2.4	50,602,169	54,798,447	68.32	5.44	2.04	69	4	103, 223, 236, 248	6.48	28.45	80.88	
	MQTL2.5	59,974,370	62,504,478	79.39	3.28	2.05	29	3	78, 81, 347	9.80	3.73	12.06	
	MQTL2.6	76,169,378	78,691,772	100.38	3.28	2.05	30	2	179, 272	6.90	22.46	85.40	
	MQTL2.7	86,054,542	95,527,021	117.70	12.28	2.05	71	3	102, 136, 181	4.66	31.81	61.40	
	MQTL2.8	128,784,834	146,796,428	178.63	23.36	2.06	209	3	37, 182, 210	2.87	34.84	32.95	61.35
3	MQTL3.1	37,791,682	48,742,705	50.77	12.85	3.04	152	6	104, 108, 232, 273, 293, 297	5.25	45.69	71.88	
	MQTL3.2	66,025,720	74,385,995	82.38	9.81	3.04	50	6	39, 105, 107, 140, 283, 291	5.19	47.07	79.16	
	MQTL3.3	86,871,013	88,157,865	102.69	1.50	3.04	15	3	82, 109, 211	5.23	18.78	92.01	
	MQTL3.4	97,685,681	106,131,178	119.58	9.91	3.04	65	7	137, 185, 237, 264, 290, 302, 351	5.48	41.13	75.91	
	MQTL3.5	114,657,635	119,157,355	137.18	5.28	3.05	36	7	138, 218, 284, 298, 349, 350, 352	5.84	41.6	87.31	
	MQTL3.6	137,024,141	140,629,030	162.90	4.24	3.05	61	3	106, 110, 139	3.71	25.83	83.58	81.64
4	MQTL4.1	24,596,837	28,858,623	35.45	9.60	4.05	167	5	41, 43,141, 1	7.09	14.54	33.98	
	MQTL4.2	40,283,042	47,483,131	52.75	1.40	4.05	101	3	64, 66, 68, 70	14.00	2.67	47.57	
	MQTL4.3	59,153,102	62,957,079	73.35	4.57	4.05	43	2	2, 6	5.87	7	34.71	
	MQTL4.4	72,716,737	85,318,974	94.93	6.94	4.05	152	5	42, 44, 84, 142, 144	8.24	8.1	14.32	
	MQTL4.5	88,977,285	10,2303,692	114.90	16.01	4.05	93	7	353, 86, 87, 88, 111, 143, 186,	7.24	22.09	27.52	
	MQTL4.6	105,229,509	107605954	127.57	3.42	4.05	37	4	292, 90, 91, 113	8.10	16.21	78.90	39.50
	MQTL4.7	111,372,475	111,722,075	134.01	0.42	4.05	20	3	93, 112, 187	5.88	15.69	97.32	
5	MQTL5.1	2,629,715	7,925,603	5.79	5.81	5.01	286	3	61, 62, 355	6.37	12.6	53.89	
	MQTL5.2	23,808,709	33,908,268	31.66	11.08	5.03	196	5	114, 116, 145, 189, 354	4.11	30.61	63.80	
	MQTL5.3	59,202,741	66,622,453	69.02	8.14	5.03	157	2	3, 146	7.85	11.55	29.52	
	MQTL5.4	94,432,700	11,2881,714	113.72	20.24	5.04	136	4	45, 188, 190, 299	5.73	39.13	48.27	
	MQTL5.5	127,342,860	128,099,416	140.12	0.82	5.04	5	5	115, 296, 363, 364, 365	8.30	18.98	95.68	
	MQTL5.6	189,380,404	204,393,016	216.00	16.48	5.08	417	6	274, 300, 301, 303, 304, 305	11.90	37.42	55.96	57.85
6	MQTL6.1	46,670,810	48,661,785	48.84	2.04	6.01	15	8	69, 148, 191, 193, 194, 195, 212, 356	5.21	17.92	88.62	
	MQTL6.2	66,263,375	69,454,792	69.53	3.28	6.01	33	6	76, 79, 147, 149, 150, 196	19.05	10	67.20	
	MQTL6.3	103,276,977	104,135,829	106.26	0.88	6.04	24	3	89, 192, 225	11.30	38.39	97.71	84.51
7	MQTL7.1	14,126,073	16,526,862	21.45	3.36	7.02	35	6	92, 154, 197, 199, 200, 213	4.57	21.28	84.21	
	MQTL7.2	22,768,199	26,105,010	34.20	4.67	7.02	43	3	72, 83, 198	12.97	9.42	50.42	
	MQTL7.3	39,555,860	43,442,852	58.08	5.44	7.02	42	7	117, 118, 151, 152, 153, 155, 157	8.83	16.38	66.79	
	MQTL7.4	55,654,009	58,940,804	80.19	4.60	7.02	14	7	226, 251, 275, 276, 277, 278, 279	2.68	12.57	63.40	
	MQTL7.5	69,054,843	69,947,993	97.27	1.25	7.02	7	6	156, 158, 219, 239, 267, 268	3.51	13.66	90.85	71.14
8	MQTL8.1	25,114,407	28,609,648	32.97	4.30	8.03	59	3	63, 202, 357	8.23	12.55	65.74	
	MQTL8.2	37,909,921	41,494,784	48.73	4.40	8.03	44	4	159, 160, 201, 369	3.52	26.55	83.43	
	MQTL8.3	53,483,704	56,718,228	67.63	3.97	8.03	19	3	67, 358, 368	6.52	10.7	62.90	
	MQTL8.4	63,285,044	64,547,894	78.45	1.56	8.03	17	2	71, 367	7.92	2.6	40.00	
	MQTL8.5	70,141,094	70,238,863	86.15	0.12	8.03	3	4	47, 49, 74, 75	7.50	12.05	99.00	63.02
9	MQTL9.1	38,809,005	48,949,576	60.19	13.91	9.03	123	3	50, 119, 203	5.16	28.37	50.97	
	MQTL9.2	49,430,724	53,039,338	70.28	4.94	9.03	321	2	289,120, 359	3.90	10.89	54.64	52.80
10	MQTL10.1	17,727,995	22,644,888	24.51	5.97	10.03	53	4	8, 214, 240, 288	3.85	37.89	84.24	
	MQTL10.2	49,556,025	53,443,418	62.53	4.72	10.03	25	4	51, 161, 163, 206	3.60	15.88	70.28	
	MQTL10.3	56,441,322	60,888,763	71.23	5.40	10.03	48	5	52, 162, 165, 166, 360	6.52	18.4	70.65	
	MQTL10.4	71,377,312	73,510,436	87.96	2.59	10.03	41	10	77, 80, 205, 220, 241, 259, 281, 285, 286, 294	5.50	54.45	95.24	
	MQTL10.5	78,134,951	80,391,616	96.24	2.74	10.04	34	5	54, 164, 361, 371, 372	4.53	14.94	81.66	
	MQTL10.6	121,431,613	129,288,759	152.21	9.54	10.07	211	2	53, 370	4.80	17.75	46.25	74.72
Total					320.01		4252	276		6.98			
Avg.					5.52		73.31	4.759			19.909	68.30	

*Chr., chromosome; CI, confidence interval; Avg., average; Phys_start, physical position start (bp); Phys_end, physical position end (bp).

The CI of individual MQTL regions was narrower than the mean CI of the original QTLs for that region. The average CI of the MQTLs was 5.52 cM in comparison with 19.91 cM for the original QTLs (**Table 1**). There was a reduction of 68.3% in the average CI of the MQTLs over the mean CI of the original QTLs, with a range from 12.1% (for MQTL2.5 at 79.39 cM) to 99.0% for MQTL8.5 at 86.15 cM (**Table 1**). The average reduction in CI from the initial QTLs per chromosome was 66.5%, with the lowest (39.5%) reduction on chromosome 4 and the highest (84.6%) on chromosome 6 (**Figure 1C**). The genetic and physical lengths of MQTLs ranged from 0.12 cM and 0.098 Mb (MQTL8.5) to 23.36 cM and 18.01 Mb (MQTL2.8), respectively. Five MQTLs (MQTL1.3, 1.6, 5.5, 7.5, and 8.5) had a physical interval of less than 1 Mb. Bins 4.05 and 1.05 harbored the maximum number (seven) of MQTLs, followed by five MQTLs in bin 8.03. The MQTLs explained from 2.9% (MQTL2.8) up to 19.1% (MQTL6.2) of the variance for *A. flavus* resistance. There was no significant difference between the average PVE (7.0%) of the MQTLs (**Table 1**) and the average PVE of the original QTLs (7.5%) (**Supplementary Table S1**).

### Genes located in MQTL positions

A total of 4,252 genes were identified in the 58 MQTL intervals based on the maize genome ZmB73_5b built in Biomercator 4.2.3 (**Table 1**; **Supplementary Table S5**). On average, 73 genes were identified in the MQTL regions, with the lowest (three) in MQTL8.5 and the highest (429) in MQTL9.2. The number of genes linked to the MQTLs varied from 72 (chromosome 6) to 1,197 (chromosome 5).

### Functional annotation of the genes in MQTL regions

**Figure 2:**
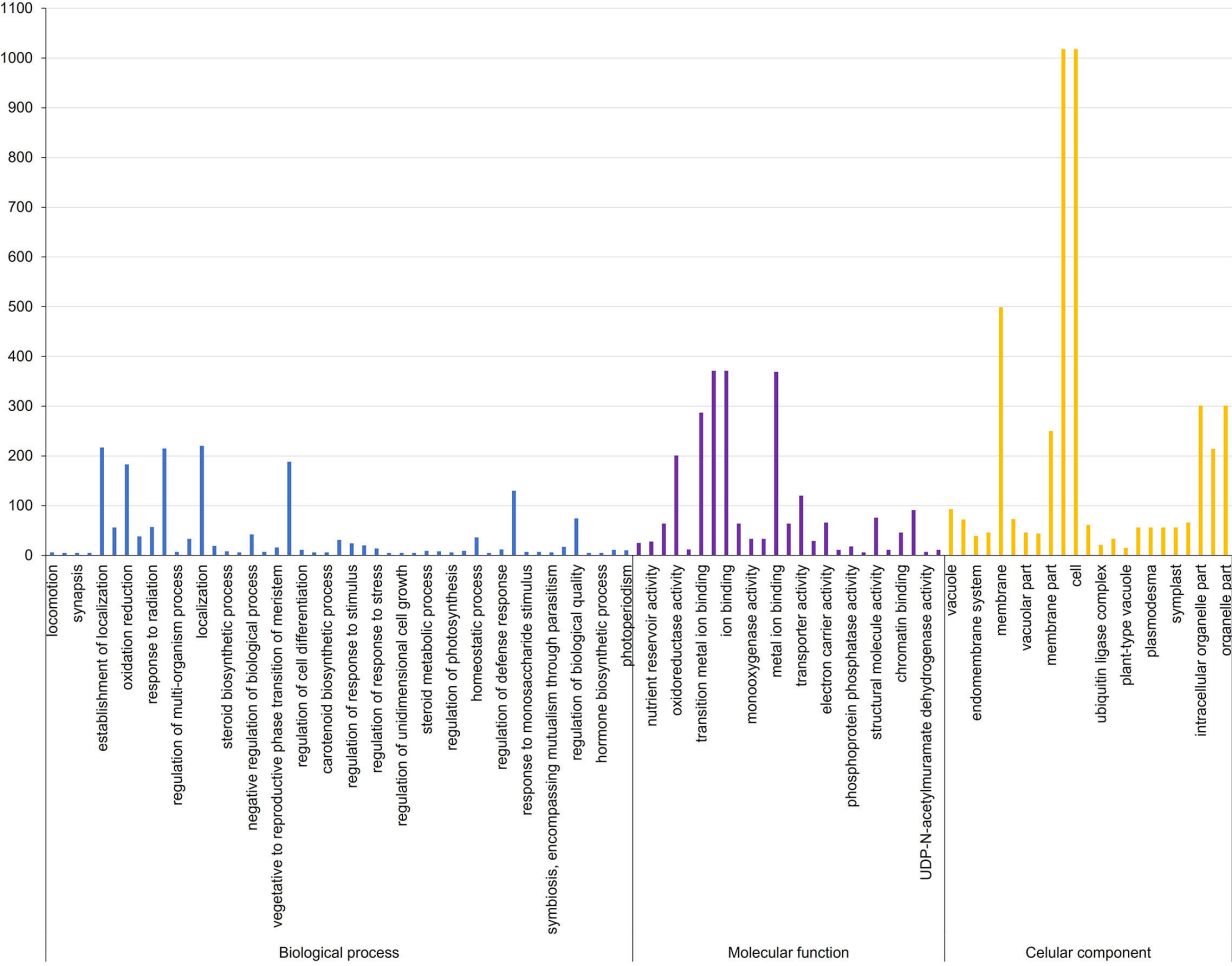
Gene ontology terms associated with biological processes, molecular function, and cellular component in *Aspergillus flavus* resistance associated 58 MQTL intervals in maize.

**Figure 3:**
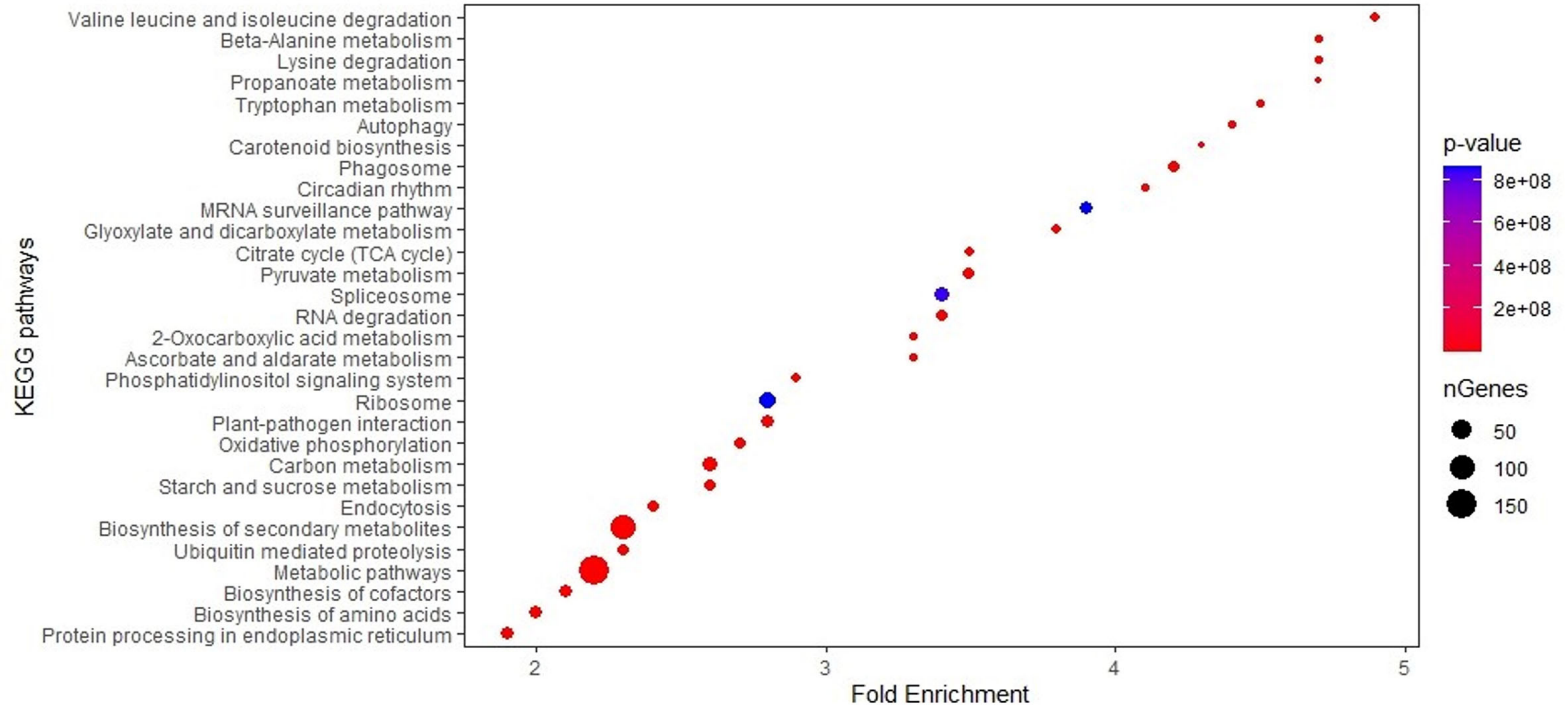
Enrichment analysis of 30 KEGG pathways for genes in 58 MQTL intervals in maize.

The biological roles of the genes in MQTL regions were ascertained by GO enrichment analysis, which showed that the highest number of genes significantly enriched in biological processes belonged to the GO term establishment of localization (217), closely followed by 215 transport mechanism and 188 in response to chemical stimulus (**Supplementary Table S6**; **Figures 2**). Significantly overrepresented GO terms associated with molecular function were related to genes involved in cation/ion binding (371), metal ion binding activity (369), transition metal ion binding (287), and oxidoreductase activity (201). For the cellular component, a significantly higher number of candidate genes were mainly related to the cell and cell part (1,018 genes), membrane (499 genes), and organelle (301 genes). KEGG analysis revealed that the MQTL genes belonged to 30 significant pathways, of which metabolic pathways and biosynthesis of secondary metabolites were the two most enriched pathways with 1,646 and 926 candidate genes, respectively (**Supplementary Table S7**; **Figure 3**).

Gene co-expression network analysis of the genes in MQTLs regions revealed that genes in four modules, such as Turquoise (445 genes), Blue (221 genes), Brown (71 genes), and Yellow (23 genes) were significantly co-expressed in maize–*A. flavus* system (**Supplementary Table S8**) with Kme values more than 0.8. In the Turquois module, out of 12 candidate hub genes with significantly high interaction (threshold >0.5), eight had functional annotations: Zm00001d003488 (UDP-glycosyltransferase 85A7), Zm00001d024444 (aldehyde dehydrogenase 26), Zm00001d017532 (RING-H2 finger protein ATL71), Zm00001d025776 (cell division control protein 48 homolog B), Zm00001d049374 (DUF295 domain-containing protein), Zm00001d025651 (fasciclin-like arabinogalactan protein 7), Zm00001d030855 (alpha zein z1D_4), and Zm00001d025672 (autophagy10) with 954, 375, 348, 306, 285, 178, 134, and 60 interactions, respectively. On the other hand, only one gene Zm00001d000004 coding for cysteine-rich PDZ-binding protein passed the threshold with 36 interactions in Blue module (**Supplementary Table S8**).

### Prediction and expression of candidate *A. flavus*-responsive candidate genes based on comparative transcriptome data

Matching analysis of the 4,252 candidate genes in MQTL regions with previously reported *A. flavus*-responsive genes (Musungu et al., 2020) identified 3,384 common genes. Comparison of the subsets of genes expressed in response to various abiotic and biotic stresses, including *A. flavus* infection (Musungu et al., 2020), in the database of both RNA-seq and microarray experiments showed that 591 genes (256 from RNA-seq and 335 from microarray) were induced only in response to *A. flavus* (**Supplementary Table S5**). Of these 591 genes, 50 showed significantly differential expressions at −1.0 ≤ Log^2^FC ≥ 1.0 (*p* ≤ 0.05) at different stages of response to *A. flavus* (Musungu et al., 2020; **Supplementary Table S5**). Furthermore, querying these 591 genes against the 3,384 genes identified 43 genes as candidates differentially uniquely expressed upon *A. flavus* infection. These 43 genes with significantly differential expressions at different stages of *A. flavus* infection (**Supplementary Figure S2**) were distributed over all chromosomes except chromosome 6, with the maximum number (14) in chromosome 5 and only one on chromosomes 1 and 8 (**Supplementary Table S9**).

These 43 genes were also queried against the gene list in qTeller, a comparative maize RNA-seq expression platform. Interestingly, 29 out of 43 genes showed expression induction or repression under abiotic stresses such as drought and/or salt (**Supplementary Table S10**; **Supplementary Figure S3**). Therefore, the remaining 14 genes were considered putative *A. flavus*-specific unique genes with no reported expression under stresses other than *A. flavus*. Eight out of the 14 genes did not have any assigned functional annotation. Two genes, Zm00001d030855 and Zm00001d003677, encode endosperm-specific proteins alpha zein and opaque endosperm 11, respectively. The remaining four genes, one each, coded for fasciclin-like arabinogalactan protein (Zm00001d025672), the mediator of RNA polymerase II transcription subunit 26b (Zm00001d040506), carboxylesterase (Zm00001d050270), and UDP-glycosyl transferase (Zm00001d013246) (**Supplementary Table S11**, **S12**).

**Figure 4:**
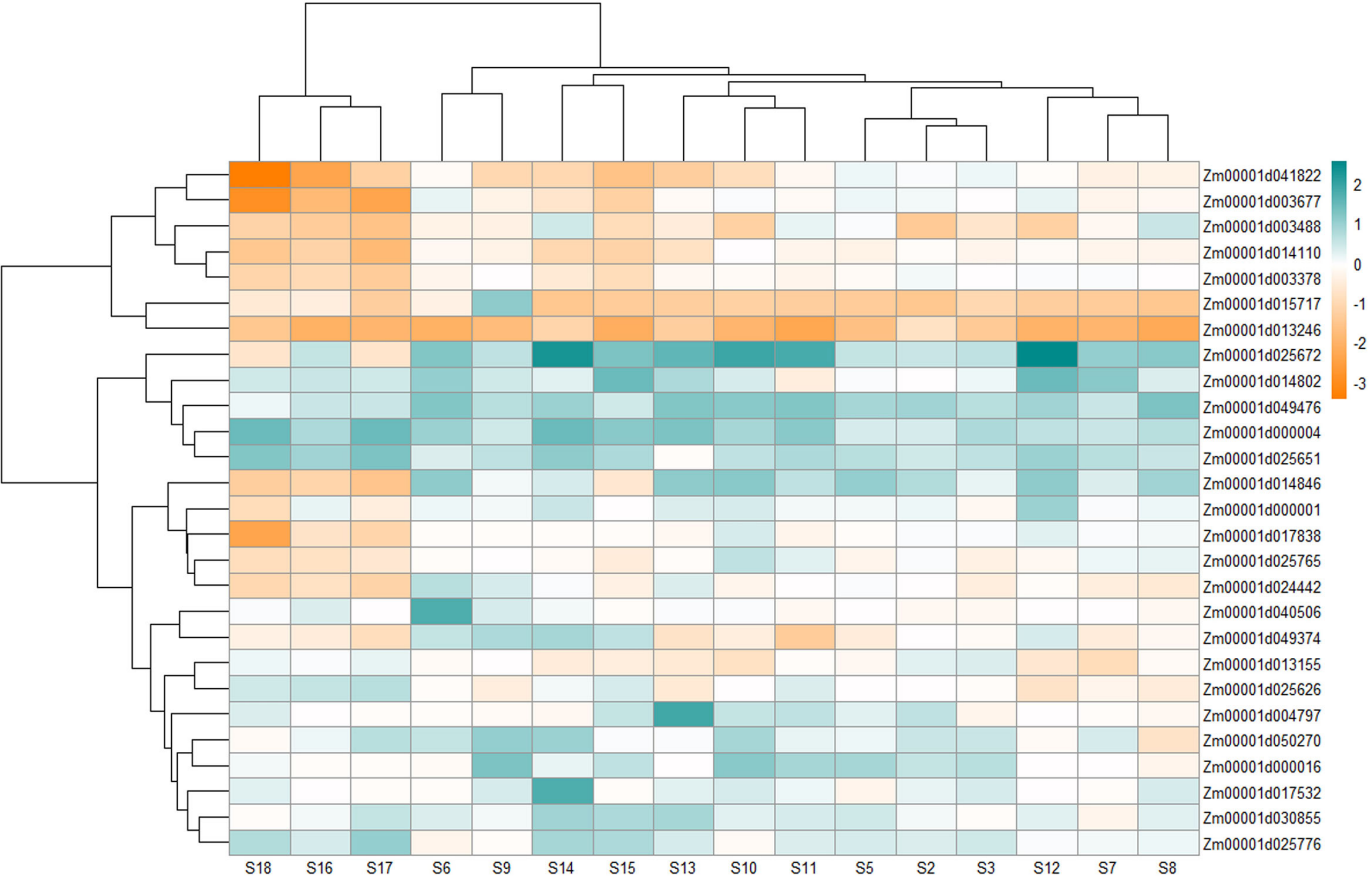
Heatmap showing the differential expression of 27 (out of 29) genes that include 14 genes uniquely expressed under *Aspergillus flavus* infection and 15 genes that were physically close between MQTL intervals and SNPs from published GWA studies. The expression values at different stages were retrieved from Musungu et al. (2020).

Alpha zein (Zm00001d030855) did not exhibit a specific expression pattern although it showed higher upregulation at most stages except at S3 and S7 where it showed downregulation by -0.12 and -0.32-fold, respectively, and at S3, S9, S16, and S18 there was no significant change in its expression (**Supplementary Table S13**; **Figure 4**). The expression was especially higher at late stages of infection. On the other hand, Zm00001d049476 for alpha-zein 19 kDa A-1 showed upregulation at all stages.

Expression of the Opaque 11 gene (Zm00001d003677), on the other hand, was repressed at most stages except for slight upregulation in S2, S5, S9, and S12. Zm00001d025672, coding for fasciclin-like arabinogalactan protein, was overexpressed with time postinfection, with its highest at S12 and S14, and then showed downregulation (S17 and S18). A putative mediator of RNA polymerase II transcription subunit 26b (Zm00001d040506) remained unchanged or maintained low upregulation with its highest at S5 and slight downregulation at S8. Zm00001d050270 (carboxylesterase) showed moderately high upregulation at all stages, with its highest at S9; however, it was also downregulated at S8. The transcript accumulation of UDP-glycosyltransferase (Zm00001d013246) was repressed at all stages postinoculation of the fungus (**Figure 4**).

### Validation of MQTLs

A total of 272 MTAs were identified from five GWAS related to *A. flavus* resistance-related traits in maize (six—Farfan et al., 2015; 222—Warburton et al., 2015; 25—Zhang et al., 2016; 11—Han et al., 2020; and eight—Bertagna et al., 2021) (**Supplementary Table S12**). Comparative analysis of the MTAs with MQTLs based on their physical intervals identified eight MQTLs (two on chromosome 2, one each on chromosomes 4 and 5, and three on chromosome 10) that colocalized with 14 MTAs, all from three GWAS (**Table 2**). Of 14 MTAs, 11 were from one GWAS (Warburton et al., 2015) that collocated with MQTL2.1, MQTL2.2, MQTL9.1, MQTL10.1, MQTL10.4, and MQTL0.6, whereas two MTAs (Bertagna et al., 2021) and one MTA (Farfan et al., 2015) co-located in the MQTL5.6 region. A total of 40 genes with functional divergence were identified within 10 kb of the GWAS-identified SNPs in the matching MQTL regions (**Table 2**). In total, 23 genes showed differential expression at various stages of infection by *Aspergillus flavus* (**Supplementary Figure S4**). Comparing these 40 GWAS genes with the 43 putative unique candidate genes in MQTL intervals showed that eight genes from GWAS were physically close (≤4 Mb) to seven genes in MQTL intervals (**Supplementary Table S13**).

**Table 2:** Forty genes within 10 kb of the SNPs associated with *Aspergillus flavus* resistance were identified from five genome-wide association studies.

MQTL/GWAS	SNP	Phys_start*	Phys_end*	Chr.*	*R* ^2^	Gene ID	Annotation
MQTL2.1	4 QTLs	13,707,326	18,150,443		6.69	–	–
Warburton et al. (2015)	S10_139505158	17,084,882	17,090,696	2	8.16	GRMZM2G076841	Tetratricopeptide TPR-1
						GRMZM2G379540	Membrane protein
						GRMZM2G379546	Uncharacterized protein
						GRMZM5G875167	Uncharacterized protein
						GRMZM2G076834	Transposable_element
						GRMZM2G379538	Uncharacterized protein
MQTL2.2	2 QTLs	38,746,143	40,967,702		6.66	–	–
Warburton et al. (2015)	S2_40498454	40,496,139	40,499,021	2	7.97	GRMZM2G151434	Hapless 8
						GRMZM2G179268	Serine-threonine kinase 2 (stk2)
						GRMZM2G179253	Uncharacterized protein
						GRMZM2G042443	Mmediator of paramutation 1
						GRMZM2G042532	Mediator of paramutation 2 (mop2)
MQTL4.1	5 QTLs	25,512,455	28,858,623		7.09	–	–
Warburton et al. (2015)	S4_26406913	26,401,952	26,408,946	4	5.60	GRMZM2G134625	NOL1/NOP2/sun family protein isoform 2
	S4_26653796	26,652,767	26,655,098		9.02	GRMZM2G003814	Alcohol dehydrogenase
						GRMZM2G101472	Uncharacterized protein
						GRMZM2G076841	Probable methyltransferase PMT13
						AC209759.2_FG006	Uncharacterized protein
						GRMZM2G111261	F-box domain-containing protein
MQTL5.6	6 QTLs	189,380,404	204,393,016		11.90	–	–
Farfan et al. (2015)	S5_197707198	197,706,593	197,707,492	5	4.85	AC209208.3_FG002	pip1b
						GRMZM2G057848	Uncharacterized protein
						GRMZM2G702166	Uncharacterized protein
						GRMZM2G057789	RING-H2 finger protein ATL1R
						GRMZM5G874697	FCS-like zinc finger 26 (flz26)
						AC207402.3_FG005	Uncharacterized protein
Bertagna et al. (2021)	S5_192698173	192,697,941	192,702,593		9.20	GRMZM2G120922	Yellow stripe-like transporter 14 (ysl14)
	S5_193181081	193,176,072	193,179,030		7.80	GRMZM5G841914	si687064f04
MQTL9.1	4 QTLs	49,430,724	53,039,338	9	4.70	–	–
Warburton et al. (2015)	S9_51159051	51,157,439	51,161,908		10.04	GRMZM2G122723	UDP-glycosyltransferase activity
						GRMZM2G039940	Uncharacterized protein
						GRMZM2G039911	Uncharacterized protein
MQTL10.3	5 QTLs	56,441,322	60,888,763	10	6.50	–	–
Warburton et al. (2015)	S10_59660506	59,660,107	59,662,383		7.66	GRMZM2G097509	Fatty acid desaturase 5
						GRMZM5G851266	Polyphenol oxidase1 (ppo1)
MQTL10.4	10 QTLs	71,377,312	73,510,436		5.50	–	–
Warburton et al. (2015)	S10_73160824	73,159,828	73,160,988		8.50	GRMZM2G107616	Uncharacterized protein
Warburton et al. (2015)	S10_73160845	73,159,828	73160988		8.51	GRMZM2G343519	Glutaredoxin domain-containing protein
MQTL10.6	2 QTLs	121,431,613	129,288,759		4.80	–	–
Warburton et al. (2015)	S10_125923329	125,923,482	125,924,714		7.85	GRMZM2G135470	Aldehyde dehydrogenase 26 (aldh26)
	S10_127808418	127,807,908	127,810,700		7.60	GRMZM2G025054	Transglutaminase 15a (tgz15a)
						GRMZM2G025366	Isocitrate dehydrogenase 3 (idh3)
						GRMZM5G835117	Uncharacterized protein
						GRMZM2G518361	Uncharacterized protein
						AC214507.3_FG001	CRM family member 6 (cfm6)
						GRMZM2G066162	Cellulase6 (endoglucanase)
						GRMZM2G066059	Autophagy 10 (atg10)

*Chr., chromosome; Phys_start, physical position start (bp); Phys_end, physical position end (bp).

**Figure 5:**
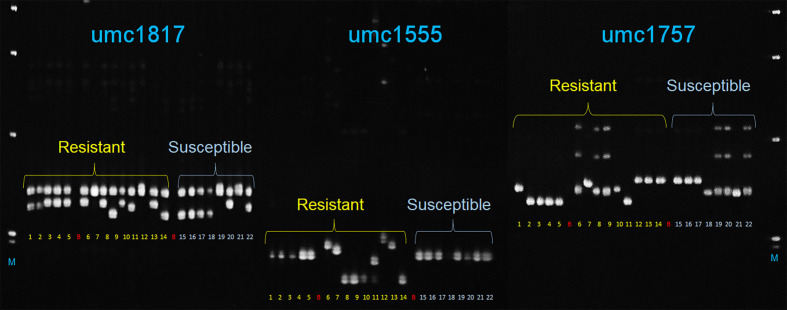
Polyacrylamide gel image showing amplicons generated by three SSR markers (umc1817, umc1555, and umc1757) linked to MQTL8.2, MQTL2.4, and MQTL4.1, respectively, with 22 maize genotypes that included 14 resistant (samples 1–14) and eight susceptible (15–22) maize cultivars. 1–12: TZAR102, TZAR103, TZAR104, TZAR105, TZAR106, MP715, MP10-127, MP04:127, MP494, MP717, MP317, MP313E, MP718, MP719, PHW79, VA35, TI73, SA212M, HI-11, A188, B104, B73; B, blank; M, 1-kb plus DNA size marker (Invitrogen Inc.).

In total, 12 SSR markers closely linked to MQTLs (**Supplementary Table S14**) were tested with 22 maize lines with known reactions to *A. flavus*. Among these, three markers, umc1555, umc1757, and umc1817 linked to MQTL2.4, MQTL4.1, and MQTL8.2 clearly discriminated between 14 and eight maize genotypes with known *A. flavus* resistance and susceptible phenotypes, respectively (**Figure 5**).

## Discussion

*Aspergillus flavus* resistance is a quantitative trait, controlled by both genetic and environmental determinants, which makes breeding aflatoxin-resistant maize variety challenging. Aflatoxin resistance is a trait with low heritability (21% for *A. flavus* infection; Busboom and White, 2004), although *H^2^* up to 63% (Mideros et al., 2014) and 74% (Maupin et al., 2003) have been reported. Several studies have identified QTLs for *A. flavus* resistance in maize (Paul et al., 2003; Widstrom et al., 2003; Busboom and White, 2004; Brooks et al., 2005; Alwala et al., 2008; Warburton et al., 2009; Mayfield et al., 2011; Warburton et al., 2011; Willcox et al., 2013; Mideros et al., 2014; Yin et al., 2014; Dhakal et al., 2016; Zhang et al., 2016). Except for Zhang et al. (2016), where the authors combined linkage-based QTL mapping with GWAS to resolve a major QTL for *A. flavus* to identify several candidate genes for resistance, constitutive, robust, and large-effect QTLs over multiple environments and across populations that are critical for deployment of markers linked to the QTLs in genomics-assisted breeding for aflatoxin resistance are lacking. Hence, consensus genomic regions that include more than one QTL surrogated by a single marker are important to incorporate one or a few alleles from the constituent QTL to improve *A. flavus* resistance. Thus far, only two studies have reported MQTLs using QTL studies on *A. flavus* resistance traits identified until 2011 (Xiang et al., 2010; Mideros et al., 2014).

We undertook a combinatorial approach of meta-analysis of QTLs reported until 2022 and gene expression data to pinpoint candidate genes (and markers) associated with *A. flavus* resistance. To identify consensus genomic regions with stable expression, we performed a meta-analysis with all 10 maize chromosomes that harbored at least six QTLs, so the possibility of poor performance of the model because of over-parameterization was ruled out (Courtois et al., 2009). The QTL dataset that we used for meta-analysis was not representative of all individual QTL mapping studies reported for *A. flavus* resistance in maize due to the lack of sufficient information and/or heterogeneity of the data needed for the MQTL analysis tool. A high-density linkage map is key to identifying stable MQTLs and selecting candidate genes for functional characterization or marker development for use in marker-assisted breeding. Consensus genetic maps created in earlier QTL meta-analysis studies (Truntzler et al., 2010; Mideros et al., 2014) rejected the existence of the same genetic map for all mapping populations. However, using markers related to the position of QTLs and (re)ordering of the markers on some genetic maps based on both genetic and physical positions, we developed an integrated high-density consensus linkage map with 5,023 markers and 2,555 cM long, much higher than 1,803 markers reported earlier (Mideros et al., 2014). High collinearity between the consensus map and the physical map obtained from the reference map B73 RefGen_v5 observed in our study was in congruence with the study of Akohoue and Miedaner (2022), which led us to project 276 out of 278 QTLs in the consensus map. Most of the MQTLs reported by Mideros et al. (2014) were identified in our 58 MQTLs over all 10 chromosomes, whereas 62 MQTLs were reported by Mideros et al. (2014) on eight chromosomes with no MQTL on chromosomes 9 and 10. The disparity in the number was due in part to some single original QTLs reported as MQTLs by Mideros et al. (2014).

As expected in a meta-analysis of QTLs, the CI (95%) of the MQTLs in our study was 3.2-fold narrower with an average of 6.3 cM (range 0.12 cM to 23.36 cM) in comparison with 19.8 cM of the initial QTLs. Previous studies in maize have shown comparable reductions of MQTL 95% CI, such as 1.4- to 36.4-fold (Akohoue and Miedaner, 2022), 3.8-fold (range 0.02 to 29.87 cM; Sheoran et al., 2022), 32% to 91% (Wang et al., 2022), and 1.89 to 14.27 cM (Kaur et al., 2021). Mideros et al. (2014) reported 8.1 cM as the average 95% CI of MQTLs, whereas three MQTLs had a 95% CI of less than 2 cM. However, we found six MQTLs, MQTL1.3, MQTL1.6, MQTL5.5, MQTL6.3, MQTL7.5, and MQTL8.5 with 95% CI <1 cM and narrower physical interval (<1 Mbp) and shorter genetic distance with at least three QTLs in those regions.

MQTLs are the target genomic regions for introgression via marker-assisted breeding programs. Thus, constant validation of identified MQTLs is needed for their effective utilization in molecular breeding. In this study, the correlations of MQTLs with MTAs obtained from GWAS were established, and the positions of MQTLs were compared with 272 MTAs associated with *A. flavus* resistance in five GWAS. Eight (MQTL2.1, MQTL2.2, MQTL4.1, MQTL5.6, MQTL9.1, MQTL10.3, MQTL10.4, and MQTL10.6) of 58 MQTLs matched MTAs from three GWAS (Farfan et al., 2015; Warburton et al., 2015; Bertagna et al., 2021), suggesting that the effects of these genomic regions on *A. flavus* resistance traits with moderate influence from the genetic background. Except for MQTL5.6, these MQTLs were different than the nine MQTLs distributed on chromosomes 1, 4, 5, 6, 7, and 8 (MQTL1.3, MQTL1.4, MQTL1.5, MQT1.8, MQTL4.3, MQTL5.6, MQTL6.2, MQTL6.3, and MQTL7.2) that were considered significant for their association with *A. flavus* resistance based on the *R* ^2^ ≥ 10 criterion. This result suggests that PVE should not be the only focus while selecting genomic regions of interest for their further validation and marker development, especially for multigenic traits such as resistance to *A. flavus* and aflatoxin accumulation.

Validation of the MQTLs was also performed using 12 microsatellite markers linked to a few selected MQTLs that were either close to the SNPs from the GWAS studies, with an average *R* ^2^ ≥ 10 or with 95% CI of less than 1 cM. The three markers, umc1555, umc1757, and umc1817, closest to MQTL2.4, MQTL4.1, and MQTL8.2, respectively, were able to distinguish the *A. flavus-*resistant and susceptible genotypes. While umc1757 at bin 4.05 of chromosome 4 collocated with S4_26406913 at ~ and S4_26653796 at ~1.1 Mb (Warburton et al., 2015), umc1555 linked to MQTL2.4 at bin 2.04 on chromosome 2 was ~10 Mb distant from S2_40498454 (Warburton et al., 2015) that was close to MQTL2.2 within the same bin. These three markers have great potential for their use in marker-assisted selection for *A. flavus* resistance, although only umc1555 and umc1757 with their discriminatory power for contrasting disease response phenotypes can be considered for the development of potential diagnostic markers. Sequences around these markers and additional sequence-based markers must be identified from large, diverse maize genotypes with known *A. flavus* resistance response to develop haplotype-specific markers for use in breeding applications.

Identification of MQTLs allowed us to efficiently search for candidate genes with potential involvement in maize–*A. flavus* interaction leading to aflatoxin toxin accumulation. A thorough comparative search of the 4,252 candidate genes in MQTL intervals with our in-house compilation of the maize transcriptome dataset identified 43 genes that were significantly differentially expressed only following *A. flavus* infection (Musungu et al., 2020). However, further interrogation of these genes against the maize gene expression database qTeller at maizeGDB showed that 14 genes were not induced/repressed under any other stress. Concurrently, 40 genes involved in various biological processes were identified as linked to the SNP markers from GWAS that collocated with eight MQTL intervals. Of the 15 genes that were close to each other between GWAS and MQTL region genes within ≤4 Mb interval, 13 had functional annotation. Therefore, these 29 genes (27 genes with functional annotation) were considered significant for their possible implications in *A. flavus* resistance. Specifically, the 13 genes out of 27 (12 in the Turquoise module and one in the Blue module) with a significant number of interactions with other genes in the network can be important candidates for further validation.

Polyphenol oxidase (PPO) such as Zm00001eb413300 plays an important role in plant defense mechanisms against biotic stresses. PPO could modify proteins by alkylating different compounds which reduces the bioavailability of proteins and prevents the digestion/absorption of nutrients in fungi (Zhang and Sun, 2021). Cysteine-rich proteins (CRPs) are involved in binding to known receptors in plants (Huang W et al., 2014). Although the signaling mechanisms and protein interactions are largely unknown, most characterized genes function as short-range intercellular signals during plant defense against pathogens (Marshall et al., 2011). A pathogenesis-related protein belonging to cysteine-rich secretory protein responded to *A. flavus* infection in maize (Hawkins et al., 2018). In cotton, a cysteine-rich kinase was differentially regulated in both pericarp and seed tissues following *A. flavus* infection (Bedre et al., 2015). The gene Zm00001eb249940 linked to MQTL5.6 was a plasma membrane intrinsic protein (PIP). PIPs are highly hydrophobic aquaporin proteins with six membrane-spanning domains that play important roles in channels that facilitate the passage of water, small solutes, and possibly other moieties through the membrane and confer abiotic stress tolerance in plants. Although the pathways involving their role in plant defense are not completely understood, the transport of H_2_O_2_, produced in response to pathogen attack by aquaporins suggests their functions in plant defense (Dynowski et al., 2008). Downregulation of some members of PIPs in soybean leaves and citrus plants upon *Pseudomonas syringae* and *Candidatus liberibacter* infection highlight their correlation with the disease development (Zou et al., 2005; Martins et al., 2015). A plasma membrane-associated protein was differentially expressed in maize upon infection by *A. flavus* (Dhakal et al., 2017).

Plant-derived fatty acids (FA) have been shown to regulate *A. flavus* colonization in the seeds by controlling pathogen development and mycotoxin production (Upchurch, 2008). Zm00001eb413200 codes for fatty acid desaturase 5 (FAD5), which is a key regulator of FA desaturation, and FADs modulate the activation of defense signaling pathways in plants, leading to PR gene expression involved in plant disease resistance (Kachroo et al., 2001).

The gene Zm00001d003378, a mediator of paramutation 1 (*mop1*), codes for RNA-directed RNA polymerase, which controls paramutation, the directed, heritable alteration of the expression of one allele at multiple loci (Alleman et al., 2006). The differential regulation of this gene in maize postinfection with *A. flavus* indicates that RdRP-mediated epigenetic changes via DNA methylation or small RNA regulation could play an important role in disease resistance responses. Poly(A)-specific ribonuclease (PARN) influences the poly(A) status of cytoplasmic mRNA in most eukaryotes. PARN might regulate the efficient translation of mRNAs that control cytosolic Ca^2+^ elevation, leading to plant responses to pathogenic fungi (Johnson et al., 2018). Plants are known to regulate defense mechanisms using polyadenylation controlled by PARN (Yang et al., 2014). The modulation of expression of maize PARN gene (Zm00001d025651) in response to *A. flavus* implies its involvement in alternative splicing of other genes involved in resistance response. Pathogen infection triggers dramatic transcriptome reprogramming, leading to a shift in plant growth and development and an immune response. During this rapid process, the mediator plays an important role in fine-tuning gene-specific and pathway-specific transcriptional reprogramming by acting as a coregulator bridge between gene-specific regulatory proteins and basal RNA polymerase II transcription machinery (Richter et al., 2022). The role of maize mediator of RNA polymerase II transcription subunit 26b (Zm00001d040506) in *A. flavus* has not been established, but the upregulation of its expression in S6, S9, and S16 stages (Musungu et al., 2020) suggests its possible involvement in resistance reactions against the pathogen in maize. A ring finger protein (Zm00001eb250960) was linked to the MQTL5.6, contained six QTLs, and was located near S5_19770198 (Farfan et al., 2015). Expression of ring/zinc finger proteins was upregulated in resistant maize cultivar MP715 (Dhakal et al., 2017) and cotton upon *A. flavus* inoculation (Bedre et al., 2015).

Cell wall-modifying genes such as UDP-glycosyltransferase (Zm00001eb382140) have been shown to be regulated by *A. flavus* infection in cotton (Bedre et al., 2015). Carboxylesterase (CXE) is an enzyme that catalyzes carboxylic ester and water into alcohol and carboxylate. CXEs in plants have been implicated in defense mechanisms. For example, a tobacco CXE suppresses the accumulation of tobacco mosaic virus accumulation (Guo and Wong, 2020). In maize, the CXE SOBER1 (Zm00001d050270) may regulate HR-mediated plant defense against *A. flavus* by possibly hydrolyzing a lipid or precursor required for HR induction, as was observed in *Arabidopsis*, where SOBER1 resulted in low phosphatidic acid accumulation in response to bacterial effector AvrBsT, causing suppression of plant immunity (Kirik and Mudgett, 2009). Cell division control protein 48 (CDC48) is an evolutionarily conserved major chaperone-like protein component of ubiquitin-dependent protein degradation pathways. In plants, CDC48 most likely contributes to protein degradation through the ubiquitin-proteasome system (Bègue et al., 2019), suggesting its role in the plant’s defense mechanism. CDC48 caused an upregulation of the expression of the NB-LRR gene *SNC1* and downregulation of the ubiquitin E3 ligase CPR1, leading to plant immunity against pathogens in Arabidopsis and tobacco (Cheng et al., 2011; Huang Y. et al., 2014), which clearly suggests the involvement of CDC48 in maize (Zm00001d025776) in resistance response under *A. flavus* attack.

Plants accumulate several reactive molecules, including aldehydes, which play dual roles in plant–pathogen interactions. Aldehydes can kill pathogens directly or act as secondary defense signaling molecules for activating durable host resistance against invading pathogens (Norvienyeku et al., 2017). At the same time, aldehydes are toxic to plant cells, and thus expression of aldehyde dehydrogenase can help scavenge excess pathogen-induced reactive aldehydes, contributing to disease resistance. Aldehyde dehydrogenase (ALDH) is a member of a group of evolutionarily conserved polymorphic enzymes that promote stress tolerance in plants (Zhu et al., 2014). In cotton, ADH was induced in the pericarp and seed tissues of cotton in response to *A. flavus* infection (Bedre et al., 2015). The gene Zm00001eb425740 codes for *atg10*, a member of the autophagy gene family. Most cytosolic proteins and organelle materials are sequestered and transported to the lysosome or vacuole for degradation via autophagy (Xie and Klionsky, 2007). Expression of the ATG genes is induced by the oxidative stress caused during necrotrophic fungal pathogen attack in plants (Lai et al., 2011) and thus could play an important role in the regulation of immunity-related programmed cell death or hypersensitive reactions in response to *A. flavus* resistance in maize. Patel and Dinesh-Kumar (2008) demonstrated that *Arabidopsis* plants with antisense suppression of the ATG6 gene showed that *RPM1* triggered a limited hypersensitive reaction in response to *Pseudomonas syringae* pv. tomato DC3000.

Zm00001d030855 and Zm00001d049476, both coding for alpha Zein proteins, showed mostly upregulation in their expression patterns in maize. Alpha Zein 19C2 precursor (P1B10) was also highly expressed after *A. flavus* infection in maize (Dhakal et al., 2017). The gene Zm00001d003677 for Opaque11 is considered the central hub of the regulatory network for maize endosperm development and nutrient metabolism (Feng et al., 2018). Novel insights are being proposed for plant storage proteins as antimicrobial proteins (De Souza Cândido et al., 2011), yet the role of storage proteins such as zein and Opaque 11 in plant defense mechanisms has not been demonstrated. They possibly regulate the *A. flavus* infection by modulating the amino acid (especially lysine) profile of the seeds.

The gene Zm00001d049374 in MQTL4.1 codes for a protein with the DUF295 domain. These plant proteins also contain an F-box domain. F-box domain-containing proteins are well established for their roles in regulating cell death and plant defense in response to pathogen responses (Den Burg et al., 2008). An F-box protein was downregulated in maize PRms RNAi lines, which suggested its possible function in PR proteins-mediated resistance response (Majumdar et al., 2017). The involvement of F-box genes in *A. flavus* was demonstrated by the upregulation of F-box3 in peanuts during the late stages of infection by the fungus (Bhatnagar-Mathur et al., 2021).

## Conclusion

Understanding the genetic basis and molecular mechanisms underlying *A. flavus* resistance is critical to developing maize varieties with improved resistance against factors affecting aflatoxin accumulation. Using a comprehensive meta-analysis of QTLs and transcriptome data, we have provided evidence of the presence of important MQTLs of possible significance in *A. flavus* resistance in maize bins 2.04, 4.05, and 8.03. Furthermore, the meta-analysis helped delimit the MQTLs to physical intervals of less than 1 Mb. Our results suggest that, although *A. flavus* resistance is a trait with a high genotype by environmental interaction effect, recurrent selection involving markers linked to the significant MQTLs as discussed above could lead to the accumulation of resistance loci that reduce *A. flavus* infection, colonization, and subsequent aflatoxin accumulation. A detailed omics study and functional validation of the identified putative *A. flavus*-specific candidate genes through genome editing or genetic engineering tools will enhance our understanding of maize—*A. flavus* interaction. Further characterization of identified MQTLs in this study to develop haplotype-specific markers and subsequent marker-assisted introgression of important MQTLs may significantly strengthen the breeding efforts for developing *A. flavus*-resistant maize cultivars.

## Data availability statement

The original contributions presented in the study are included in the article/**Supplementary Material**. Further inquiries can be directed to the corresponding author.

## Author contributions

NB - conceptualization, data curation and analysis, funding acquisition, investigation, original manuscript draft preparation. ES - investigation, data curation and analysis, review of manuscript draft. AP - data curation and analysis. KR - planning, funding acquisition, experimental materials, review of manuscript draft. All authors approved the final manuscript.
