# Supplementary material for: Comprehensive meta-analysis of QTL and gene expression studies identify candidate genes associated with Aspergillus flavus resistance in maize
Source: Front Plant Sci. 2023 Jul 18;14:1214907. doi: 10.3389/fpls.2023.1214907 (PMC10392829; doi:10.3389/fpls.2023.1214907)
Supplement: Supplementary file 1 [file DataSheet_1.pdf]

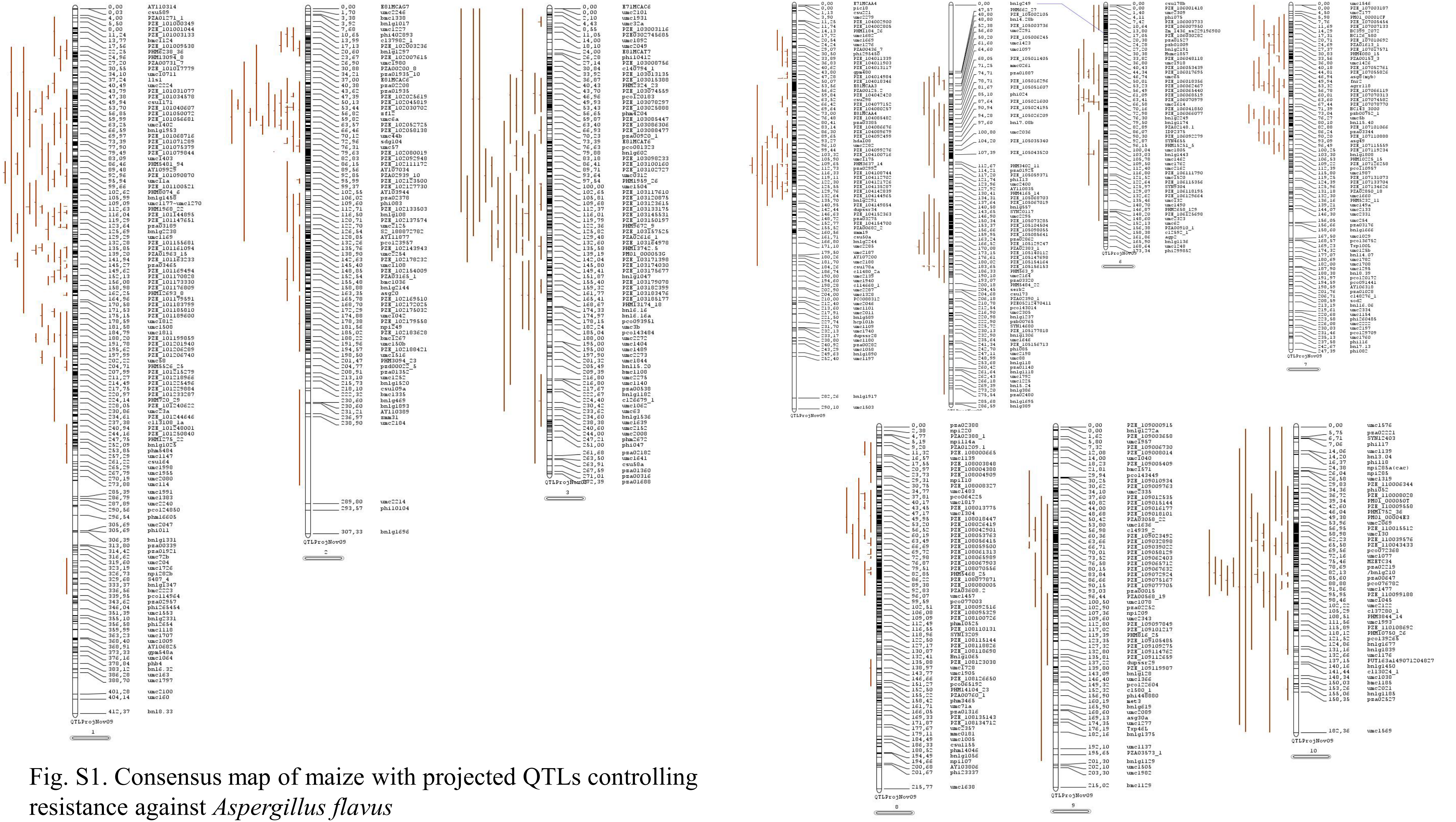

Fig. S1. Consensus map of maize with projected QTLs controlling resistance against *Aspergillus flavus*

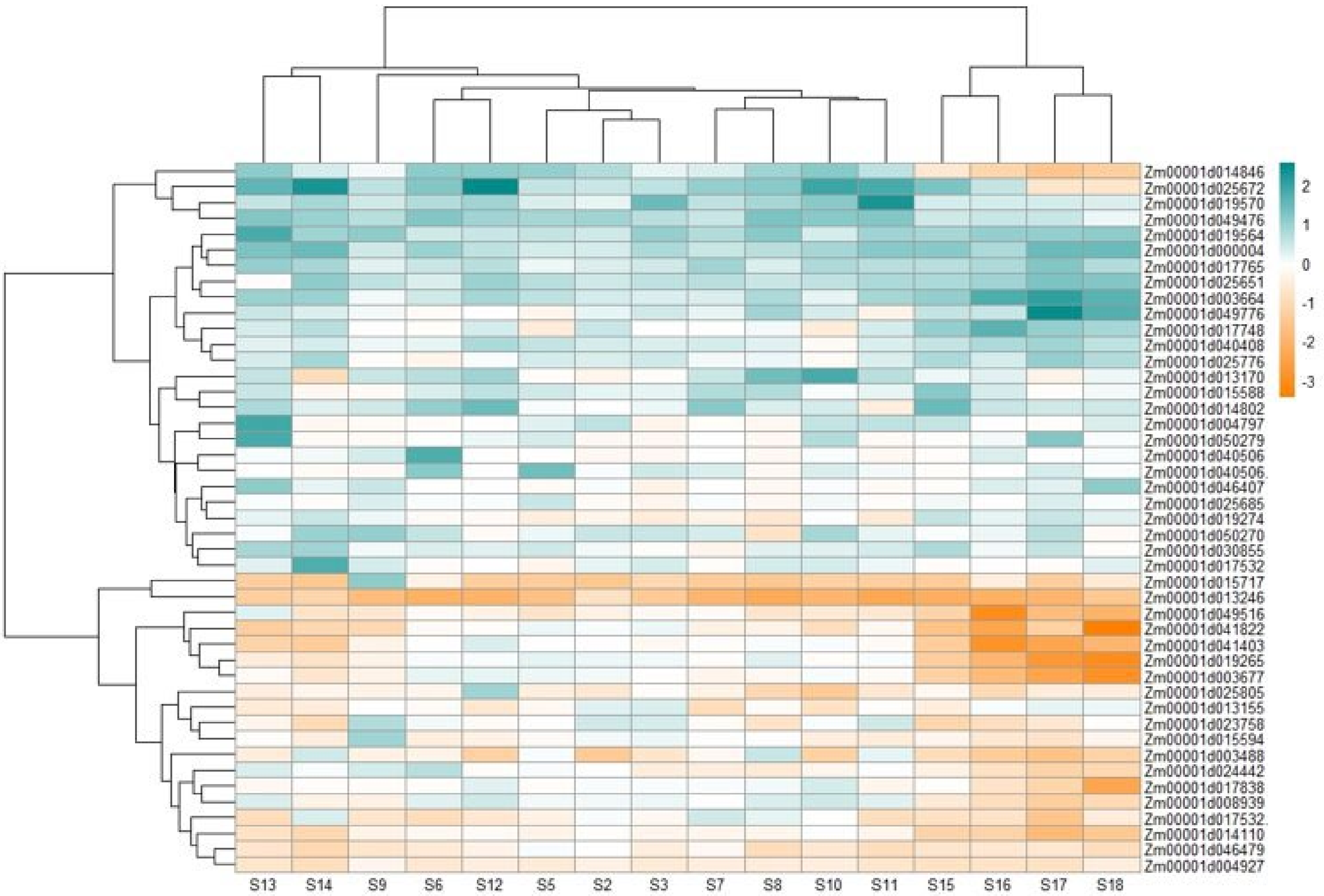

Fig. S2. Heatmap showing differential expression of 43 putative *Aspergillus flavus* specific genes in MQTL intervals at various stages of *Aspergillus flavus* infection (Musungu et al. 2020). These genes did not have any match with published genes expressed under other stresses

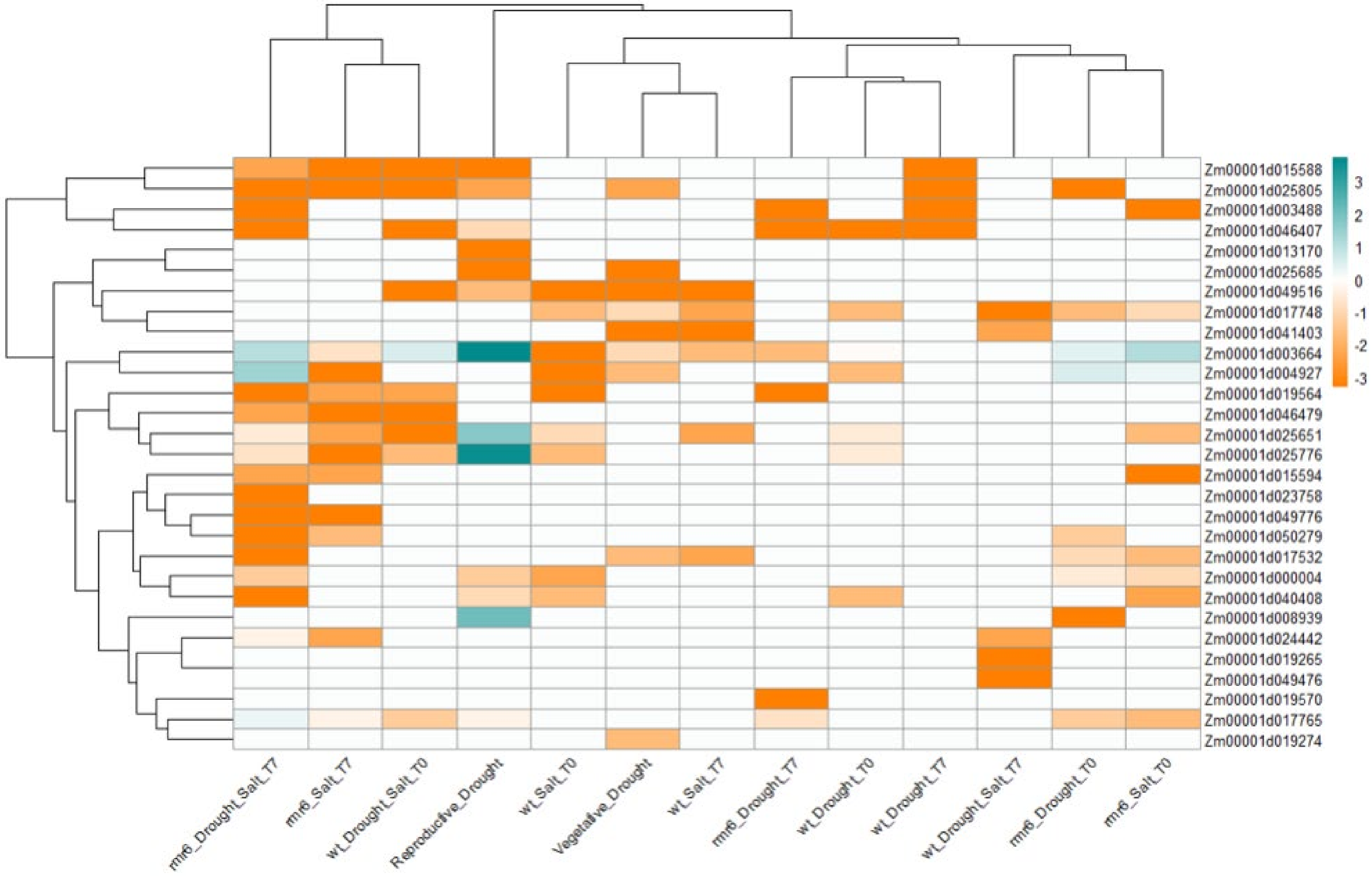

Fig. S3. Heatmap showing expression of 29 genes under drought and salt stresses in qTeller database. These genes belong to the set of 43 putative *Aspergillus flavus* specific genes in MQTL intervals that did not have any match with genes expressed under other stresses (Fig. S2)

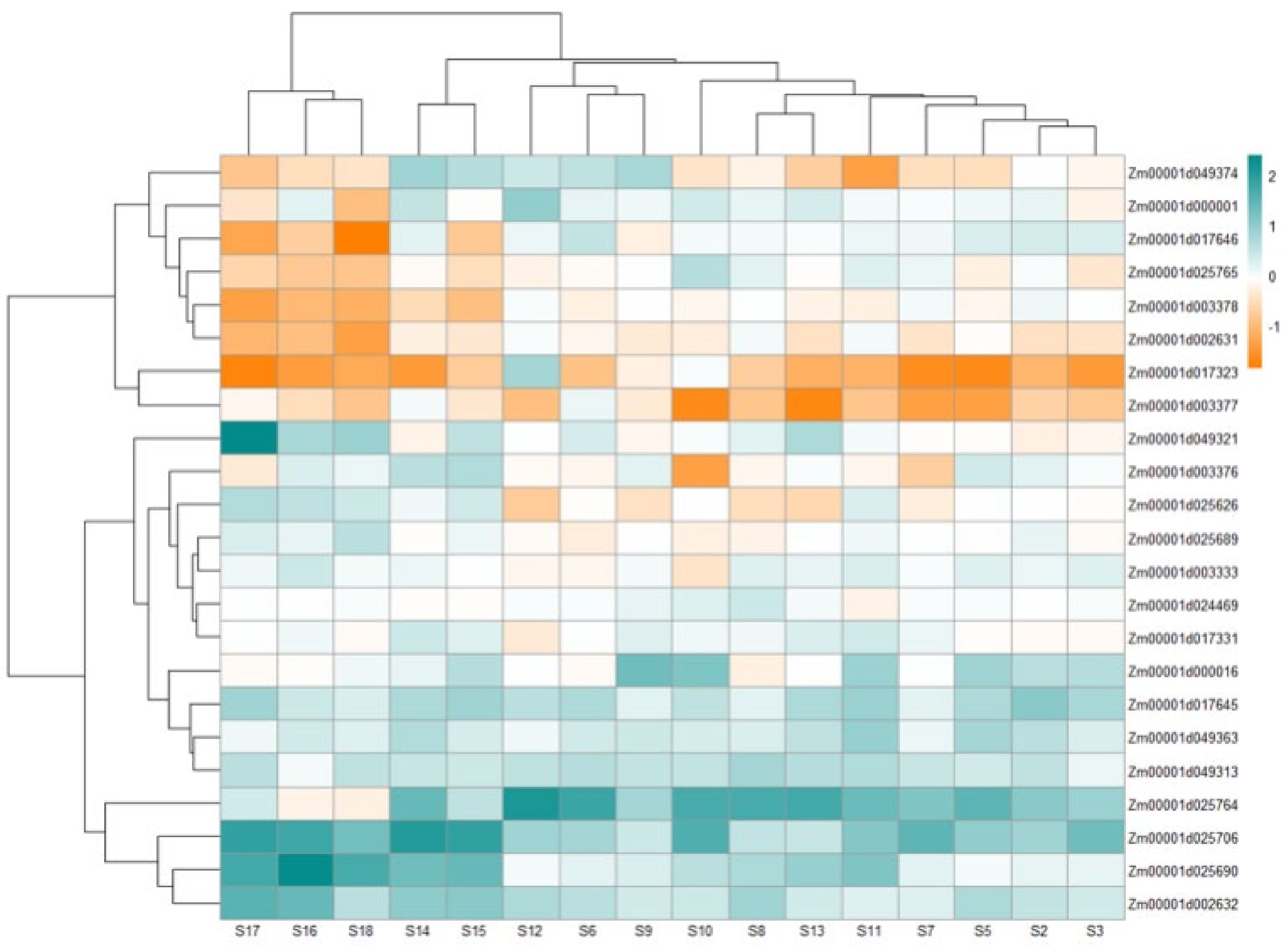

Fig. S4. Heatmap showing differential expression of 23 candidate genes flanking 10 Kb to GWAS SNPs at various stages of *Aspergillus flavus* infection
